# The Brainarium: An Interactive Immersive Tool for Brain Education, Art, and Neurotherapy

**DOI:** 10.1155/2016/4204385

**Published:** 2016-09-06

**Authors:** Romain Grandchamp, Arnaud Delorme

**Affiliations:** ^1^Laboratoire de Psychologie et NeuroCognition, Université de Grenoble, Grenoble, BSHM, 1251 av Centrale CS40700, 38058 Grenoble Cedex 9, France; ^2^CNRS, UMR 5105, Grenoble, France; ^3^Centre de Recherche Cerveau et Cognition (CerCo), Université Paul Sabatier, Pavillon Baudot, Hopital Purpan, BP 25202, 31052 Toulouse Cedex 3, France; ^4^CNRS, UMR 5549, Toulouse, France; ^5^Swartz Center for Computational Neuroscience, Institute of Neural Computation (INC), University of San Diego California, La Jolla, CA 92093-0559, USA

## Abstract

Recent theoretical and technological advances in neuroimaging techniques now allow brain electrical activity to be recorded using affordable and user-friendly equipment for nonscientist end-users. An increasing number of educators and artists have begun using electroencephalogram (EEG) to control multimedia and live artistic contents. In this paper, we introduce a new concept based on brain computer interface (BCI) technologies: the Brainarium. The Brainarium is a new pedagogical and artistic tool, which can deliver and illustrate scientific knowledge, as well as a new framework for scientific exploration. The Brainarium consists of a portable planetarium device that is being used as brain metaphor. This is done by projecting multimedia content on the planetarium dome and displaying EEG data recorded from a subject in real time using Brain Machine Interface (BMI) technologies. The system has been demonstrated through several performances involving an interaction between the subject controlling the BMI, a musician, and the audience during series of exhibitions and workshops in schools. We report here feedback from 134 participants who filled questionnaires to rate their experiences. Our results show improved subjective learning compared to conventional methods, improved entertainment value, improved absorption into the material being presented, and little discomfort.

## 1. Introduction

This century has been marked by the development of new brain imaging techniques, which have allowed us to better understand how our brain functions when we experience different mental states. The brain appears as a key integrative organ where a variety of inputs are simultaneously processed and combined: exteroceptive stimuli, that is, stimulations coming from the external world, proprioceptive inputs that provide body state information, or interoceptive inputs such as thoughts, emotions, and other inner experiences [1]. This processing is the result of lifelong learning, shaping, and adaptation of our neural system through our interaction with the world [2].

With the discovery of some of the core processes underlying brain electrical activity, we have found a new way of looking at the brain of living beings, obtaining insights on the functioning of their perceptual and inner spaces. When groups of several thousand neurons in the brain, sitting at close distances from each other and oriented in the same direction, are firing synchronously, their joint electrical activity adds up and generates an electrical field that is strong enough to be captured on the scalp. As a result of technological developments in electronics, signal processing, and computer science, we are now able to record different electrical brain rhythms with millisecond precision and process this activity in real time by placing electrodes on subjects' head. The technique of electroencephalography (EEG) is now widely used both in fundamental and in clinical research, as well as a diagnostic tool in clinical environment. In addition to basic research and clinical applications, EEG rhythms have been recently used to control computers in real time. In Brain Machine Interface (BMI) or Brain Computer Interface (BCI) [3–5] characteristic patterns of EEG activity during specific mental activity are mapped to a given computer command. Some BCI systems allow controlling a mechanical device, a graphical interface, or a video game using thoughts only. Subjects may voluntarily learn to retrain specific brain EEG patterns in order to correct pathological activity of the brain. This specific range of application is called neurofeedback or neurotherapy [6–8].

When EEG began to be recorded in the 1930s, researchers realized that several typical rhythms could be distinguished in the brain electrical activity recorded at the surface of the scalp. The first “brainwave” was identified by the father of electroencephalography, Berger [9]. It was denominated using the first Greek letter, alpha, and became the “alpha” rhythm, which is a brain rhythm that oscillates at about 10 cycles per second (10 Hertz). This rhythm is particularly active when a person is awake, resting with his/her eyes closed or while relaxing [10, 11]. Using alpha brainwaves to create or modulate sound and/or music has been pioneered by Lucier [12] as recently as 1965. Later in 1969, Kamiya showed that it was possible to voluntary control the alpha brain rhythm and modulate audio feedback in real time [13].

Following technical and theoretical progresses in neuroscience, computer science, and signal processing, EEG signals have recently been used in new ways [14, 15]. With the development of affordable and user-friendly EEG systems, the last few years have seen an increasing number of art projects using brain electrical activity as an input or way to produce or modulate artistic content such as computer graphics, animations, music, and choreography. Several performances have been created around the concept of music generation using brainwaves [16–19]. The Global Mind Project (http://www.globalmindproject.com/) is an example of such an artistic project. This system allowed for audio-video rendering of brain data, which, when combined with live interactive performance, has helped further develop new interactive artistic productions. According to Clarke, an Honorary Fellow in the Department of Culture and Communication at the University of Melbourne, “drawn together in a coalescence of self and technology, the artists connected to the EEG headsets are presented as both automata – self-operating machines – and intentional, self-activating beings, that have the ability to affect and be affected by the on-screen imagery generated” [20].

Another recent realization developed by a team of Rensselaer Polytechnic Institute students is Yehuda Duenyas' Infinity Simulator which involves control of a 3D automated rigging system using specific brainwave patterns [21]. This device led to the creation of the Ascent project (http://theascent.co/), a live-action, participatory theatrical experience that combines mind-control and levitation via an automated custom-built lifting platform system.

Our system uses similar ideas with the important addition of an immersive environment. This is the first time to our knowledge that* real time EEG* recordings are being displayed in a* full dome immersive environment* allowing* direct spatialization* (spatial transposition) of brainwave data. One of the main originality and strengths of our system is also the brain metaphor regarding the shape of the device. Among developed applications, it allows projecting EEG topographic activity directly on the dome surface of a planetarium as if viewers were standing at the centre of the brain, looking up at electrical brain activity projected on the scalp.

The “Brainarium” (originally “Cerveaurium” in French) was initially designed to present neuroscience concepts in a fun, attractive, and interactive way for educational and entertainment purposes, by mixing art and science. In the method section of this paper, we will first present the general concept and architecture of the system in order to outline and illustrate its general functioning, describe a first performance that was designed for the Brainarium, and detail the specific implementation of the performance using open source software platforms. In the Result and Critical Reception section, we then mention the context in which our device has been used during exhibitions at museums and during “The Brain's Awareness Week” and present data on audience's experience in the Brainarium. In Discussion, we finally introduce potential extension and further development of our system in the fields of education, entertainment, arts, and more specifically its possible benefits in clinical applications such as neurotherapy.

## 2. Methods 

### 2.1. Concept and General Design

**Figure 1:**
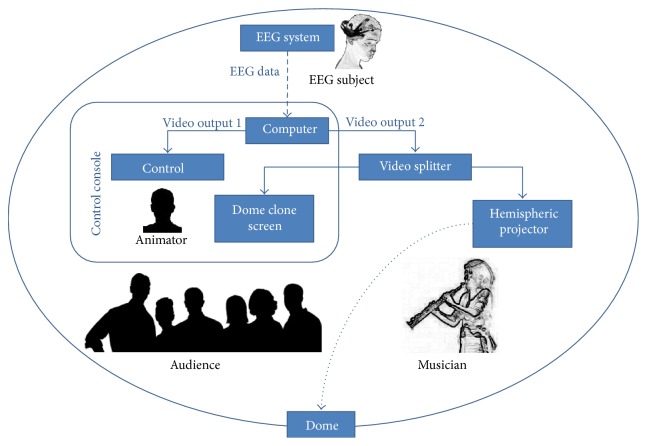
General principle of the Brainarium. EEG is recorded using the Emotiv headset and sent to a computer that computes brain rhythm activity in real time and projects it on the planetarium dome.

**Figure 2:**
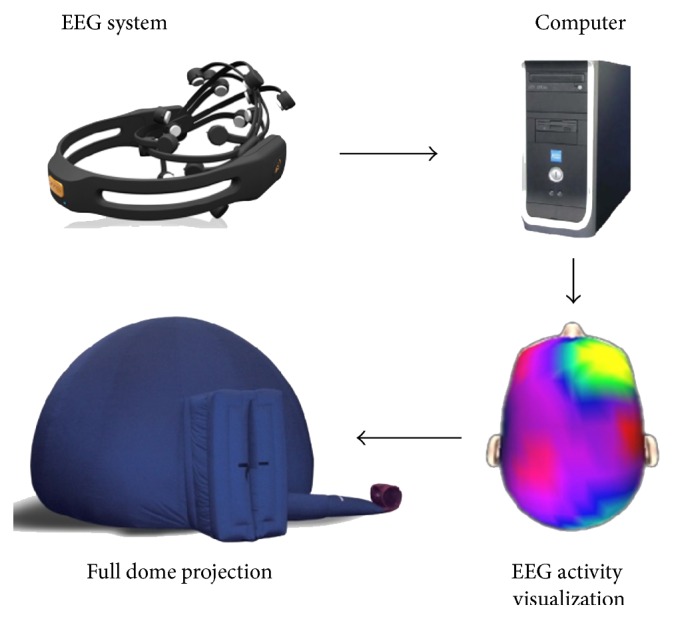
Flow chart of the different modules of the Brainarium.

Figures 1 and 2 summarize the architecture of the system and the different hardware it is comprised of. EEG signal is acquired on a person present in the dome. As shown in these figures, we used the Emotiv Epoc headset (Emotiv, Inc.), which includes 14 metal electrodes recording electrical brainwaves on the surface of the scalp at a frequency of 240 Hz (240 samples per second), but any EEG system compatible with BCI software can potentially be used. The signal is then transmitted, using a wireless connection, to a computer. This processing unit handles the signal processing part and calculates the control signals, which will be used to drive multimedia contents. Visual representations are finally projected onto a planetarium dome via a video projector equipped with a hemispherical lens (the system can be adapted to project on a hemispheric mirror which will reflect the image on the dome surface rather than directly project on the dome using a hemispheric lens). The system can be upgraded to multiprojector full dome systems but the main advantage of using a transportable inflatable dome and monoprojector hemispheric projection system is that it decreases the overall cost and allows an itinerant use. The computer display adapter should have two video outputs in order to allow simultaneous control of the different software on one screen and output to the video projector for the dome. In addition, a video splitter was used to send the video signal to a second screen so that the person driving the performance could see what was being projected.

If the system is used in the context of an art and science performance, brain electrical activity may be recorded from a member of the audience, an organizer of the projection, or an artist who participates in the event. Our device opens a wide range of possibilities among which we have integrated and used the following for a first performance:

- Interaction with animation in computer graphics through electrical brain(s) activity(ies).
- Visualization of a brain rhythm (Alpha rhythm) associated to the suppression of visual input when the subject closes his eyes or relaxes.
- Real time presentation of topographies of brain electrical activity.
- Interactive presentation of brain's structures on a 3D brain model.

After presenting the general implementation of the system, the next sections will be devoted to description of each one of these applications.

### 2.2. General Implementation

Our set-up is based on combining a hemispheric projection system such as the one used in a planetarium, a hemispheric projection surface, and a brain computer interface system. Since every functional block of the system is modular, various solutions may be developed depending on budgetary constraints and available material. As we are writing this paper, the cost of building such a system could range from about US$5,000 to about a hundred thousand dollars when using research grade apparatus; the intermediate set-up we present here costs about US$40,000 although we also provide suggestions on how to build a similar system for a lesser amount.

For projection, we used a transportable planetarium system, which comprised a Digitarium® Delta Portable Digital Planetarium System [22] and a Digitalis™ Portable Dome [23] which has a diameter of 7 meters. However, both hemispheric projection systems and projection surfaces may be made at a lesser cost using custom made tools [24–26]. We implemented a low cost solution to replace the Digitarium Delta Portable Digital Planetarium System. This solution is composed of four parts: a full HD video projector (Acer H7531D), a condensator (Rodenstock TV Heligon 75 mm F/D = 1.1 can be replaced by a classical 50 mm with F/D = 1.4 combined with a +4 diopters lens as well), a 50 mm 45° mirror mount (Skywatcher), and a fisheye lens (Peleng 8 mm f3.5 fisheye lens). The dome we used was made of a thick fabric inflated by a powerful fan. This solution makes it more convenient to transport and set up the system compared to a rigid dome solution. However, this method has the drawback of having to leave the fan turned on in order to keep the dome inflated. Even if the sound of the fan is not covering the sounds played inside the dome, it still creates a distracting background noise.

The control console was composed of a classic personal computer equipped with a dual screen graphic card powerful enough to handle HD projection and two LCD monitors. One of the LCD monitors was used to control the demonstration. On the second graphical output, a video splitter was used to send the display signal to both a control LCD monitor and the video projector. For the EEG signal acquisition, the research edition package of Emotiv Epoc headset was used [27]. Emotiv Epoc is a wearable EEG “headset” composed of 14 gold-plated electrodes. In order to record electrical signals generated by the brain, each electrode is covered by a small felt-based pellet that acts as a bridge between the electrode and the scalp. These pellets have to be soaked in a saline solution, of water mixed with salt, which allows electrical conduction from the skin to the metal electrode through the pellet. The advantages of using this system are that it is relatively low in cost compared to clinical or research oriented devices. It is also wireless, fast, and easy to set up and provides some level of spatial resolution since it has 14 electrodes. However, clinical or research EEG systems with better signal quality can be used if available. Dry active electrodes would be the most adapted for such a system as they provide acceptable signal quality with a minimum preparation time but they are still expensive to date compared to the Emotiv Epoc solution.

The complete list of software used to run the system is depicted in Table 1. Except from the Emotiv software suite (the basic software package provided with the Epoc headset by Emotiv), the software used to do signal processing and visualization is all part of the open source community. For the fractal application, the software package “Mind Your OSC” was used to collect data from Emotiv Control Panel software and send it as an Open Sound Control (OSC) [28] stream to visualization software. The interactive fractal video was displayed using the vvvv software (https://vvvv.org/), a graphical programming environment for easy prototyping and development. The vvvv software application is designed to facilitate handling of large media environments with physical interfaces, real time motion graphics, audio, and video that can simultaneously interact with many users. The freely available OpenVibe software [29] was used for signal acquisition, signal processing, and visualization of the EEG data in the context of the EEG topography application. Finally, the 3D brain model application has been developed using Blender (https://www.blender.org/), a free open source 3D content creation suite, and rendered by its embedded real time full dome plugin [30]. We are making available all additional plugin and software developed for our application under an open source license [31].

### 2.3. Performance Design and Implementation

In this section we detail technical implementation of each application used for the different phases of the original performance designed for the Brainarium.

#### 2.3.1. Brain-Controlled Animation of Fractals

**Figure 3:**
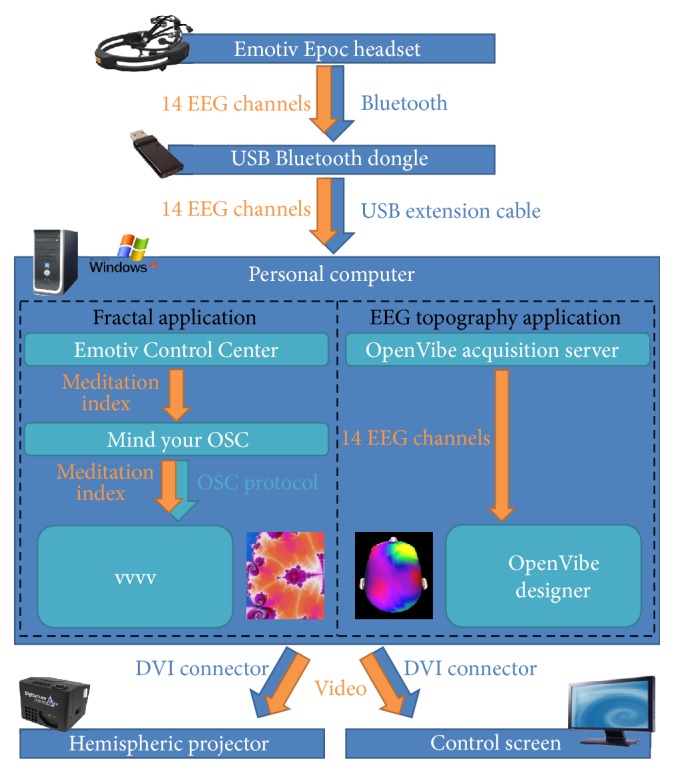
Set-up of the Brainarium for two different application examples: the fractal dynamical zoom application and the EEG topography application.

This application is an example of live interaction.  Figure 3 depicts the general architecture of the hardware and software for this application.

We first placed the EEG cap on the subject's head. Ideally, the subject's alpha brainwaves should be large compared to the overall electromagnetic noise. Since individual brains show different electrical rhythmic activities, some subjects can exhibit low amplitude alpha oscillations and this might make it more difficult to process the signal without the use of advanced artifact rejection techniques. Due to time limitation between sessions, we often asked a preselected person with known high amplitude alpha rhythm (i.e., easily observable on the signal trace) to be the subject.

After checking electrodes contact quality and signal quality, a calibration step lasting approximately two minutes is performed in order to evaluate some statistical features of the signal of interest's amplitude for the selected subject such as its mean and standard deviation. We used the index “Meditation” provided by the Emotiv Control Panel as the control signal. Since it has not been made public by Emotiv, we do not have the exact formula used to compute this index out of the raw EEG signal. However, it is known to be positively correlated with the alpha rhythm and relaxation. Emotiv indexes result from a statistical analysis based on a large normative database collected of many subjects and are therefore already normalized. However, a calibration procedure is still used in order to adapt the system to subject's specific statistics. In our case, we used the standard deviation and the mean value of the “Meditation” index over the calibration period as a reference value to tune the feedback set-up.

**Figure 4:**
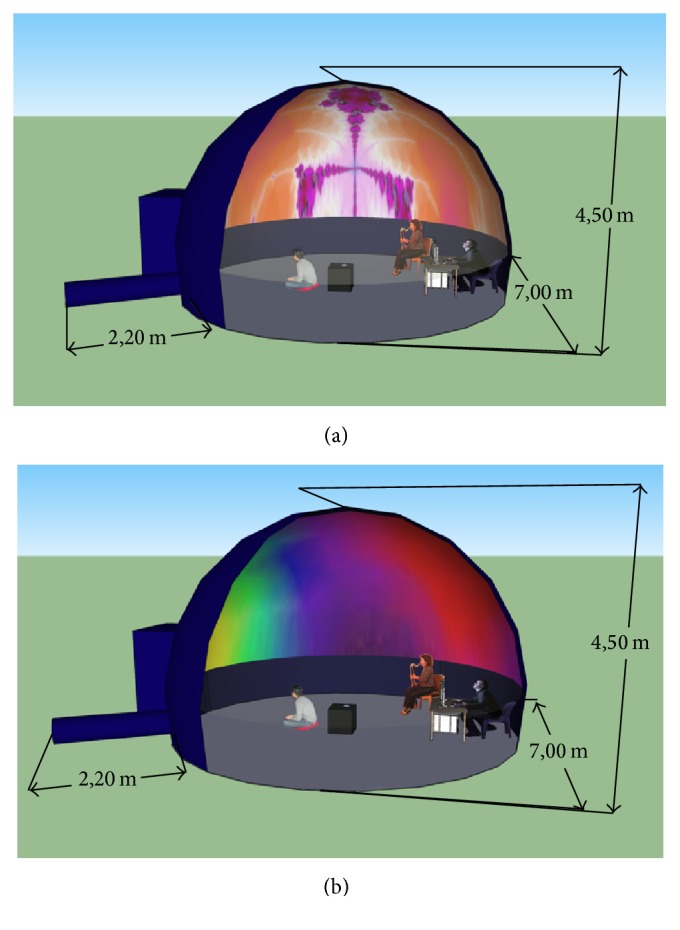
Brainarium represented as a 3D model with exact dimensions (the public is not shown on the rendering). (a) shows the projection of fractals and (b) shows the projection of subject's scalp topographies.

During the first minute of calibration we asked the subject to keep his eyes open and during the second minute we ask him to keep his eyes closed. Even if the subject has already performed the experiment, it is important to repeat the calibration step since EEG features widely vary throughout the day and from one day to another. “Meditation” values are calculated for both the eyes-closed and the eyes-open period and are used to calibrate the system to allow balanced behaviour of the visual feedback animation. Once calibration is performed, the session starts with the video feedback being projected on the dome and the audience enters the dome. In the meantime, a professional musician is improvising based on the visual display. This allows creating a complete interactive feedback loop between the subject wearing the EEG device and the musician (Figure 4(a)). The musician uses inspiration of what he sees on the dome to play music and adapt it. Furthermore, he can engage in an interplay with the wearer of the EEG and can try to induce changes in what is displayed.

The “Meditation” measure controls the display projected on the dome. We used a zoom into Mandelbrot's ensemble fractal as visual feedback. More details about the video used are given in following paragraphs; we focus here on the interaction configuration. The speed and direction (forward or backward) of the zoom depends on the brainwaves of the subject wearing the EEG cap. The system was set up so that the animation was played forward, as if diving or moving forward into the fractal, when the current alpha wave amplitude generated by the subject was over its mean level. By contrast, when the current alpha wave amplitude was lower than its mean level, the animation was played backward, as if travelling away from the fractal. The speed of the animation was modulated by the difference between the current value of the alpha “Meditation” wave amplitude and its mean amplitude; that is, zooming becomes faster as the current value is further away from the mean. As a result, a “Meditation” value equal to the mean value would result in a static image.

The shapes projected on the dome are fractals. A 2D fractal is a mathematical expression, which may be represented as a 2D image. We choose to use fractals because, in addition to their aesthetic dimension, more and more research is showing that certain aspects of brain activity or even its own structure share some features with fractals [32–35]. Because fractals are based on mathematical expressions, there is no theoretical limit to the resolution of fractal images making it possible to zoom in on a small portion of the image and expanding it indefinitely. Another feature of fractal images is that their structure is preserved regardless of the “zoom.” Finally, fractal images are self-similar representations. If the appropriate “zoom” is applied to a fractal image, the same image may be found again. An interesting feature resulting from the use of a fractal animation is that it produces an immersive tunnelling effect.

Fractal images presented in the Brainarium were made dynamic by zooming in or out in the fractal image. The animation used in the Brainarium was “a precalculated journey into the heart of the Mandelbrot fractal set” (http://www.hd-fractals.com/), which is named after Benoit Mandelbrot, the mathematician who studied and popularized it [36]. The video used in our demonstration features a 2^∧^760 zoom in the Mandelbrot fractal set and it was produced by Teamfresh (http://www.hd-fractals.com/), an independent production company which specialized itself in rendering fractal animations. We used a commercially available High Definition version of the animation. The fractal video control application has been specifically implemented for this project using vvvv, a graphical programming environment for easy prototyping and development (https://vvvv.org/). We have made the vvvv patches developed for this application freely available [31].

#### 2.3.2. EEG Real Time Topography Application

Specific software for this application can be seen in Figure 3. During the second part of the performance, participants observe EEG raw brainwaves, followed by their representation as topography or how EEG brainwaves are distributed on the surfaces of the scalp. These EEG topographies may be likened to topographies used in elevation maps for hiking. Instead of representing the terrain elevation on the Earth surface, colors represent the strength of a specific brainwave at different locations on the head surface. In our case, we focused on brainwaves in a frequency band ranging from 8 to 12 Hz called the alpha band. Alpha brainwave amplitudes vary quickly in time and space and this dynamic may be rendered as animated colored maps on the dome. The topography is represented using either classic 2D spherical projection or an interactive 3D head model from OpenVibe software [29]. Using this set-up, participants may observe that when the subject closes his eyes, alpha wave amplitudes increase on the part of the dome that represents the back of the head. The part of the brain that is activated is called the occipital region, which is a brain area largely devoted to visual processing. When this region does not process visual information, that is, when the subject closes his eyes, alpha waves tend to increase in this brain area. Another way to increase alpha wave amplitude over the entire brain is to ask the subject to enter a deep relaxation state but this requires more training from the subject and this is more difficult to achieve in a single session: we have succeeded to perform the second part of this demonstration with only a few subjects. While the brain dynamic is shown on the dome, a musician is simultaneously playing his instrument, trying to help the subject to go into deeper relaxation states and simultaneously giving him auditory feedback about his relaxation state using his own interpretation of ongoing EEG patterns (Figure 4(b)). We have made available under an open source license the OpenVibe software scenario we developed to display alpha wave topography [31].

#### 2.3.3. Neuroanatomy Using a 3D Interactive Brain Model

After the two interactive real time EEG sessions, the last part of our demonstration interactively showed different parts of the cortex in human brain volume. Despite the BCI being not involved in this part, we still want to describe it briefly to keep the description of the system's features complete. On the basis of gross topographical conventions, the cortex can be classified into four lobes: the temporal lobe, occipital lobe, parietal lobe, and frontal lobe. The system developed using Blender Game Engine (https://www.blender.org/) allows manipulating the 3D model in order to show the different lobes and introduce some of basic neuroanatomy concepts. We implemented rotation around different axis, zooming in and out for projection of these 3D models on the dome. The 3D models are rendered using the “Blender embedded full dome plugin” to compensate deformation due to the dome-specific projection lens and surface. We are making publicly available the Blender file we developed [31].

## 3. Results and Critical Reception

The innovative aspect of our project was to combine real time brain electrical activity visualization tools with an immersive full dome environment. Participants were seated inside the space enclosed by the projection dome, which induces a special atmosphere and feeling. In addition, scientific and artistic content interactive display exploited the analogy between the shape of the projection space and the near spherical shape of the brain (see Methods). What participants heard was not necessarily limited to what was being played inside the dome, as the material used for the projection surface was not soundproof. Nevertheless, acoustic properties of the dome were specific to its hemispheric shape, and this tended to enhance participants' experiences.

The Brainarium was inaugurated during “The Brain's Awareness Week,” an event organized every year in all large European cities. For a week, series of exhibits are set up to present to the general public the latest advances in brain research. During “The Brain's Awareness Week” 2013, we performed more than 17 sessions demonstrating the Brainarium to more than 200 visitors. Following this encouraging start, our demo was also presented in Paris during the Cognitive Sciences Forum in the “Couvent des Cordeliers,” at the Medical School of Paris, where it proved to be a very popular animation with more than 180 visitors in one day. Our project was also featured on the most popular newspaper of South-West France (6 million readers), “La Depeche,” and also mentioned on local radio stations. It is now regularly requested for performances in more and more cities across France and Belgium, for workshops in primary and secondary schools, and for various national events such as the French National Science Week.

A questionnaire was filled in by participants after the performance to collect their feelings and how their experience in the Brainarium compares to traditional conferences and lectures they attended.

This questionnaire allowed us to collect demographic data about participants, on four closed questions with Likert scales, and an open text field where subject could give us their feedback freely. The first question asked the participant if he or she feels this type of demonstration promotes learning and memory compared to a conventional conference. Answer was given on a 5-point Likert scale ranging from 1 (“not at all”) to 5 (“a lot”). The second question asked the participant whether it was more entertaining than a traditional conference or course. Answer was given on a 5-point Likert scale ranging from 1 (“less entertaining”) to 5 (“more entertaining”). Question three addressed whether participant was more or less absorbed by the presentation on the 3D dome compared to a presentation on a conventional rectangular screen. Answer was given on a 5-point Likert scale ranging from 1 (“less absorbed”) to 5 (“more absorbed”). Finally, the fourth question asked if the participant felt discomfort (i.e., if he felt dizzy) due to the presentation on the 3D dome. Answer was given on a 5-point Likert scale ranging from 1 (“not at all”) to 5 (“a lot”). We collected data on a total of 134 participants in two distinct performance places, during four different days. 52 participants were men and 82 were woman with an average age of 30.4 ± 17.3 years old across all participants (minimum age was 7; maximum age was 80).

**Figure 5:**
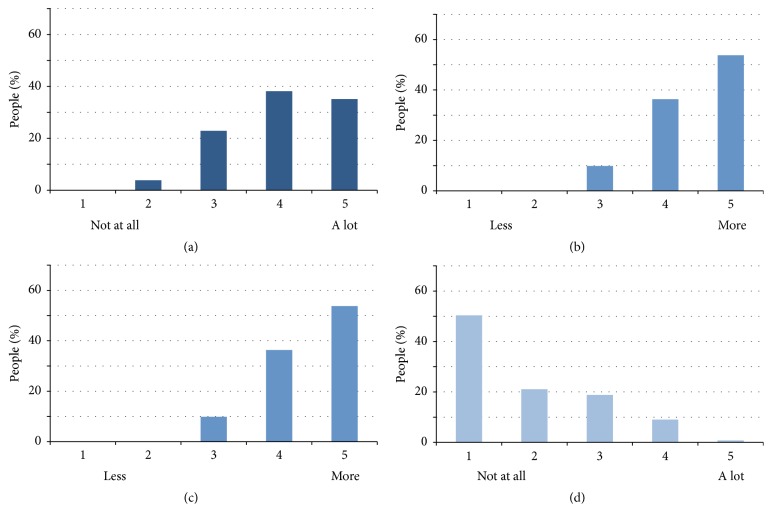
(a) Improved learning. (b) Entertainment. (c) Absorption. (d) Discomfort.

Results from the questionnaire are shown in Figure 5. Our results show improved subjective learning compared to conventional methods, improved entertainment value, improved absorption into the material being presented, and little discomfort with no participant experiencing strong discomfort.

## 4. Discussion

Planetarium domes have previously been used to display various contents. However, to our knowledge, this is the first time that real time EEG data is being shown in such an environment. Our demonstration appeared to arouse some level of popular success and seemed to provide participant with a new type of interactive experience. Thus, we have made all the tools we developed available in the public domain for anyone interested in reproducing our demonstration.

In the following sections, we will focus on four domains of application in which the Brainarium may potentially be used and further developed: education applications, entertainment applications, art applications, and immersive neurofeedback applications.

### 4.1. Education and Training Applications

The current Brainarium set-up already provides educational material to explain some basic concepts in Cognitive Sciences. We are currently exploring the possibility of showing content using stereoscopic projection methods, with the goal of providing an even more intense immersive experience to the public.

We currently focus on porting two classical BCI applications to the dome environment and developing pedagogical materials. The first application involves visualizing brain electrical activity related to emotion. Recent studies have reported that it is possible to differentiate emotional reactions and states using EEG in real time [14, 37]. When the participant wearing the EEG headset is experiencing a given emotion, an appropriate dynamical pattern reflecting the subject's emotion would be shown on the dome. The second application involves visualizing brain electrical activity associated with real and imagined body movements. Execution or mental visualization of body movement gives rise to typical brain rhythms [38]. These rhythms are recorded at the scalp surface and may be used to control visual display or even robotic devices. Moreover, results brought by fMRI studies on these domains can be shown to complement the explanations, showing brain areas and brain processes involved. The ultimate goal is to use the interactive and immersive dimensions to create and stimulate curiosity, attention, and interest in order to serve pedagogical purposes.

### 4.2. Entertainment Applications

The Brainarium could potentially be used as an immersive environment for BCI based games. BCI appear as a potential new way to gain control over a video game or a virtual world [39, 40]. Several EEG products specifically developed for BCI games have recently been made available to the general public in the form of commercial games (Star Wars Force Trainer and Mindflex by Matel, Inc.) and video games (Mindout: http://www.mindoutgame.com/, Free [41]). Several game studios have even specialized to solely design BCI games (MindGames: http://mindgames.is/, Dreams of Danu: http://www.dreamsofdanu.com/).

Immersive environments such as hemispheric projection surfaces have been already used for video games (e.g., with Blender full dome compatible Game Engine) [30], but never in conjunction with BCI systems. Moreover, it has been pointed out in a previous study by Lalor et al. [42] that subjects report that the multimodal feedback, such as the visuoauditive feedback delivered by the Brainarium, is useful in learning to control the game by suggesting that immersion increases sensation and therefore provides a more enjoyable game experience.

However, the engagement in the task of controlling the game using brainwaves might be too demanding and might degrade game experience. Nelson et al. [43] showed that concentration on the BCI task interacted with the feelings of presence in a virtual reality environment. However, they report as well that over time BCI control became more automatic for subjects as their brain adapts to the device, which allowed them to be gradually more absorbed by the virtual reality environment and feel more present. This description varies from what most subjects who experience the same virtual environment without BCI report: initially participants feel a high sense of presence which gradually drops as they realize the limitations of the virtual environment [44].

But what does the dome bring compared to a classic head mounted virtual reality device such as 3D goggles? An experiment studied the experience of users in an immersive device called the Cave [45], a room in which the user is presented with high-resolution stereo-pair images projected in real time on 3 walls and the floor, which provides an experience similar to a dome environment. They compared the experience of users in several environments: no immersion, head mounted 3D goggles, and the Cave. Subjects rated the Cave as providing a more immersive experience than all other conditions. Subjects also reported that the Cave was more comfortable than the head mounted goggles. There are numerous potential causes of visual discomfort when viewing stereo displays [46]. One of them is the vergence-accommodation conflict, that is, small amounts of left/right asymmetries, which is potentially present in all conventional stereo [47]. These results argue in favour of dome or room based systems for producing highly immersive environments.

### 4.3. Art Applications

More and more exploratory work using digital media and interactive devices are emerging on the art scene, leading to the relatively new field of interactive art. This developing genre of art usually has the public providing input in order to determine some parts or characteristics of the created content. Interactive art provides a ground for dialogue between the artist and the public through the potential of actions or reactions, introducing either intentional or passive ways to act upon the artwork.

The Brainarium is specific in the sense that the participant brainwaves are the source of interactivity. The artist may modulate multimedia artwork projected on the dome based on participant brainwaves. As mentioned for the education application parts, the artist may be able to extract subject's emotion and adapt the art forms being shown on the dome. Our system finally opens up the possibility to live coparticipation involving one or several participants wearing EEG headsets.

### 4.4. Medical Applications Using Immersive Neurofeedback

Neurofeedback is a type of brain computer interface application used in clinical environments to help to treat pathological traits [48–51]. Neurofeedback is being used to treat neuropsychological pathologies, epilepsy, ADHD, addiction, and depression [6, 52, 53], and to improve performance (stress management, creativity, attention and focus, and control of impulsivity [7, 8, 54–57]). The idea behind neurofeedback is that pathological mental states generate abnormal brain rhythms. By training patients to control their brain rhythms and suppress the pathological ones, it might be possible to treat specific mental pathologies. Note that neurofeedback is not yet widely accepted in the scientific and medical communities although recent neuroscientific works indicate some level of clinical efficacy and a bright future for this discipline [58–62].

Recent research results brought evidence that, in the context of neurofeedback training, immersion tends to improve training efficiency compared to classic feedback on a 2D screen [63]. As stated by Lécuyer et al. [64], virtual reality (VR) technologies provide motivating, safe, and controlled conditions that enable improvement of BCI learning. As reported in a recent review by Pfurtsheller et al. [65], a realistic virtual and immersive environment enhances the feeling of presence, task performance, and also cortical activation [66–68]. Studies indicate that the more game-like and engaging neurofeedback applications often resulted in a better performance [69, 70]. Subjects report the games are more stimulating and that multimodal immersive feedback is useful [42].

Previous studies have used virtual reality goggles with neurofeedback [63] but neurofeedback has never been performed in immersive environments like the one we are presenting here. Immersive environments could potentially offer numerous other benefits to patients, such as reduced training time, improved classification accuracy, increased sense of immersion and presence in an artificial setting, and reduced boredom or fatigue [71]. Finally, in the context of a therapeutic neurofeedback session, the dome environment provides a unique environment for enhanced intimacy between the patient and the therapist.

In the specific field of emotion regulation, fMRI neurofeedback recently brought very promising results [72–76]. However and despite the difficulty of recording subcortical regions of the brain involved in emotion generation, result obtained with EEG recordings [14, 15, 37] could be extended and refined in order to benefit from the high temporal resolution of the EEG and target in particular cortical areas involved in emotion monitoring and regulation [77]. Independent component analysis and source reconstruction methods could potentially be used to improve EEG spatial resolution and signal to noise ratio. Cannon et al. [78] showed that limbic lobe and hippocampal activity can be recorded and visualized using LORETA during affective memory recall. In another study, Cannon et al. [79] showed that it was possible to learn to self-regulate activity in anterior cingulated gyrus, an area of the brain known to be involved in both cognitive and affective processes. ICA neurofeedback and LORETA neurofeedback are indeed possible in an immersive set-up such as the Brainarium. Following recent developments in the field of virtual reality technology, several studies argued in favour of several benefits from using virtual reality in treatment of various pathologies or disorders related to emotions such as anxiety disorders (for a review see [80]). Bringing together BCI and VR could potentially help to not only better monitor and therefore optimize the therapy, but also give birth to new therapeutic techniques.

## 5. Conclusion

We described the first interactive system allowing real time spatialized visualization of electrical brain activity in a brain-like shaped immersive environment. This device was initially intended to deliver scientific knowledge using a pedagogical medium at the crossing between art, science, and technology. Its modular architecture allows extending and adapting it to various implementation solutions leveraging the costs to different contexts of deployment. This innovative concept can be further developed into a rich variety of applications in educational, entertainment, art, and medical domains.

## Figures and Tables

**Table 1:** List of hardware and software applications required for setting up the Brainarium.

Software	Operating system	Function
Emotiv Control Center	MS Windows	Acquire EEG data and transmit it to the software “Mind Your OSC”

Mind Your OSC	MS Windows	Receive data from Emotiv Control Center and transmit them to vvvv using OSC protocol

vvvv	MS Windows	(i) Receive OSC data packets from “Mind Your OSC” (ii) Calibrate the system (iii) Compute the video speed (iv) Display hemispheric video

OpenVibe Acquisition Server	MS Windows or Linux	Acquire EEG data and transmit it to OpenVibe Designer

OpenVibe Designer	MS Windows or Linux	(i) Collect data from OpenVibe Acquisition Server (ii) Process EEG signal (extract alpha frequency band) (iii) Compute and display real time EEG topography

Blender	MS Windows or Linux	Display a 3D brain model in the Game Engine with a full dome display mode
